# MIPs and Aptamers for Recognition of Proteins in Biomimetic Sensing

**DOI:** 10.3390/bios6030035

**Published:** 2016-07-18

**Authors:** Marcus Menger, Aysu Yarman, Júlia Erdőssy, Huseyin Bekir Yildiz, Róbert E. Gyurcsányi, Frieder W. Scheller

**Affiliations:** 1 Fraunhofer Institute for Cell Therapy and Immunology, Branch Bioanalytics and Bioprocesses (IZI-BB), Am Mühlenberg 13, Potsdam D-14476, Germany; 2 Institute of Biochemistry and Biology, University of Potsdam, Karl-Liebknecht-Strasse 25-26, Potsdam D-14476, Germany; aysu.yarman@yahoo.de; 3 Turkish-German University, Faculty of Science, Molecular Biotechnology, Sahinkaya Cad. No. 86, Bekoz, Istanbul 34820, Turkey; 4 MTA-BME “Lendület” Chemical Nanosensors Research Group, Department of Inorganic and Analytical Chemistry, Budapest University of Technology and Economics, Szent Gellért tér 4, Budapest H-1111, Hungary; julia.erdossy@gmail.com (J.E.); robertgy@mail.bme.hu (R.E.G.); 5 Department of Materials Science and Nanotechnology Engineering, KTO Karatay University, Konya 42020, Turkey; bilgi@karatay.edu.tr

**Keywords:** biomimetic recognition elements, aptamers, molecularly imprinted polymers, chemical sensors, aptasensors, in vitro selection, SELEX

## Abstract

Biomimetic binders and catalysts have been generated in order to substitute the biological pendants in separation techniques and bioanalysis. The two major approaches use either “evolution in the test tube” of nucleotides for the preparation of aptamers or total chemical synthesis for molecularly imprinted polymers (MIPs). The reproducible production of aptamers is a clear advantage, whilst the preparation of MIPs typically leads to a population of polymers with different binding sites. The realization of binding sites in the total bulk of the MIPs results in a higher binding capacity, however, on the expense of the accessibility and exchange rate. Furthermore, the readout of the bound analyte is easier for aptamers since the integration of signal generating labels is well established. On the other hand, the overall negative charge of the nucleotides makes aptamers prone to non-specific adsorption of positively charged constituents of the sample and the “biological” degradation of non-modified aptamers and ionic strength-dependent changes of conformation may be challenging in some application.

## 1. Introduction

**Figure 1:**
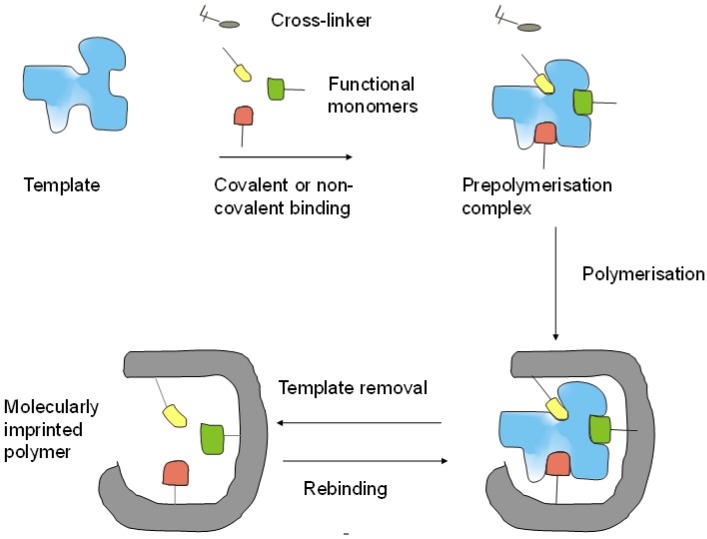
Simplistic workflow of molecularly imprinted polymers (MIP)-preparation.

The selective molecular recognition is the very essence of many biological processes; therefore, the idea to substitute antibodies and enzymes by “biomimetic recognition elements”, either on the basis of nucleic acids or fully synthetic polymers has fascinated a large community of scientists from polymer chemistry, biochemistry, organic chemistry, material sciences and bioanalysis. These efforts involved the rational reconstruction of individual biological units (e.g., bioinspired chemical synthesis of the active sites of enzymes), as well as the development of universal concepts for generation of synthetic receptors. Certainly, the second approach seems more attractive for many reasons and, therefore, this paper aims at presenting probably the two most established concepts in this respect, i.e., the molecular imprinting of polymers and the in vitro selection of aptamers for selective recognition of proteins. A broad spectrum of “selective sorbents” and later of synthetic polymers with specific binding sites—so called molecularly imprinted polymers (MIPs)—has been developed based on early concepts from the 1930s [1,2] and 1940s [3] (Figure 1). This involves polymerizing functional monomers in the presence of a target compound (analyte for analytical applications) to result in synthetic polymers that bear the imprint of the target, i.e., the imprinting process generates binding sites for a given target. The prerequisite of a successful molecular imprinting is the interaction of the target through covalent (pre-organized approach) [4,5] or non-covalent (self-assembly approach) [6,7,8] bonds with the chemically active moieties of the functional monomers in the pre-polymerization mixture. This arrangement is fixed in the subsequent polymerization of the functional monomers and reaction with a cross-linker. After polymerization, the template molecules are removed, providing binding sites that are ideally complementary in size, shape and functionality to the template, thus, the template preferentially rebinds to these sites.

**Figure 2:**
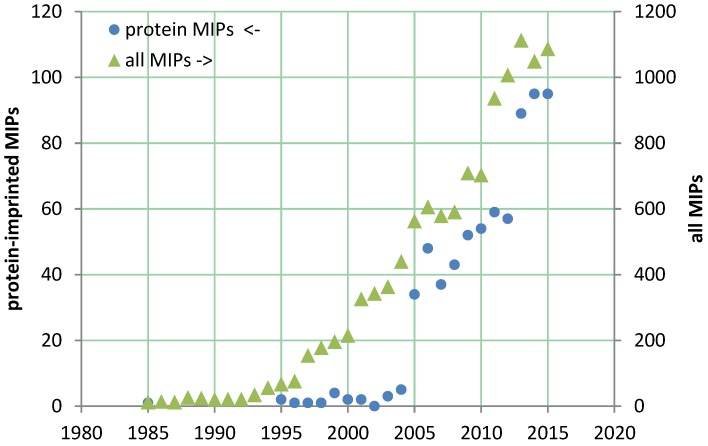
Number of publications on protein-imprinted (circles) and all molecularly imprinted (triangles, right axis) polymers, until the end of 2015 (generated by means of MIPdatabase [16]).

The majority of publications and patents describe MIPs for the recognition of low molecular weight substances. In spite of the exponentially growing number of publications on MIPs (Figure 2), their commercial application is still limited to a few examples in separation techniques [9]. The development of MIPs for proteins had a slow start from 1995 [10], with only a few papers up to 2004, but it exploded in 2005 [11,12,13,14] and covers at present almost ten percent of all publications on MIPs [15]. Protein-MIPs have great potential in both diagnostic and therapeutic applications. Since the generation of MIPs for bio-macromolecules is substantially more complex than for small targets this field is still in the phase of development research.

**Figure 3:**
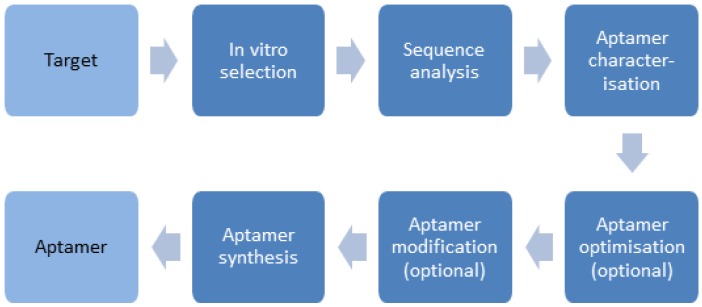
Schematic workflow of aptamer generation.

In 1990, three independent research groups reported on an in vitro method, called now SELEX (Systematic Evolution of Ligands by EXponential Enrichment), for selection of nucleic acids binding a target molecule with high affinity and specificity [17,18,19], i.e., so-called aptamers. The term “aptamer” derived from the Latin word aptus and the Greek word meros signifying “parts that fit” was introduced to describe their original selective binding property. Aptamers are primarily short, single-stranded nucleic acids (ssDNA or ssRNA) with a typical length of 20 to 120 nucleotides [20], but peptide aptamers can be also generated [21,22,23,24]. Nucleic acids can fold into manifold complex three-dimensional structures as a function of their sequence and the conditions of their solvents [25,26,27]. These structural varieties enable very specific and high-affinity binding to a target molecule as a result of a combination of hydrogen bonds, π–π stacking and electrostatic interactions. Aptamers have already been selected against a multitude of targets, such as small organic molecules [17,28], nucleic acids [29], amino acids [30,31], antibiotics [32,33], peptides [34], proteins [19,35], bacteria [36,37], viruses [38], or even whole living cells [39,40]. The broad selection of successfully applied target molecules underscores the great potential of aptamers as it was possible to develop aptamers with dissociation constants (K_D_ values) in the picomolar to low nanomolar range [27,41], comparable and sometimes even better than those of monoclonal antibodies. The generation of high affine and high specific aptamers requires a well-defined process of several steps (Figure 3).

**Figure 4:**
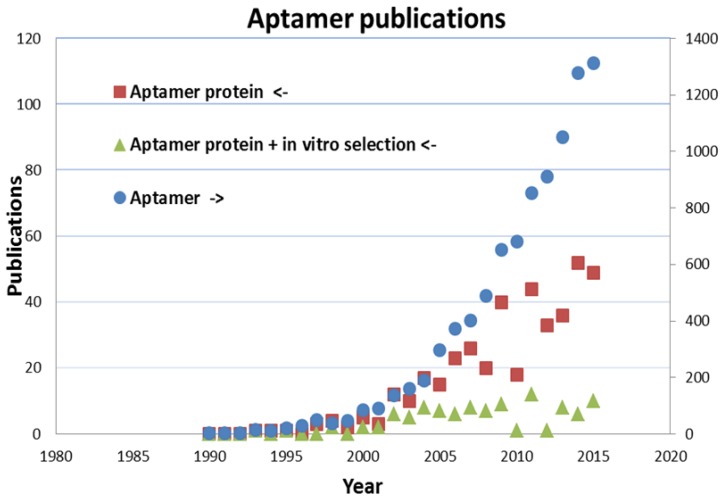
Number of publications subdivided to the search terms ‘aptamer protein’ (squares), ‘Aptamer protein + in vitro selection’ (triangles) and ‘aptamer’ (circles, right axis), until the end of 2015 (generated by means of Scopus [43]).

Tuerk and Gold [19] described the SELEX method and its first application to select a RNA aptamer against the bacteriophage T4 DNA polymerase. This result was only possible by the establishment of the chemical synthesis of high diversity nucleic acid libraries. Former works by Spiegelman [42] also isolated RNA ligands against Qβ RNA polymerase but only by evolution experiments. Since the introduction of SELEX twenty-six years ago, new RNA and DNA aptamers are generated more and more against different proteins, particularly against those of therapeutic relevance. The number of all aptamer-related publications is exponentially growing and its current rate (over 1000 publications per year) is comparable to that of MIP-related publications (Figure 4). This rapid increase is mainly fueled by the increasing interest in aptamer-based biosensors.

The measuring process by biomimetic sensors can be divided into two essential steps:

- molecular recognition of the analyte by the aptamer or MIP, which is determined by the corroborative effect of the composition/sequence and spatial arrangement of the respective biomimetic elements.
- generation of a measurable signal as result of the interaction of the target-loaded biomimetic recognition element with the transducer.

Both aspects and representative examples of sensors for proteins are presented in this article.

## 2. Preparation and Performance of MIPs for the Recognition of Proteins

### 2.1. Building Blocks of MIPs

Proteins are typically made up by the total spectrum of the 20 natural amino acids. Whilst chemists have a comparable arsenal of building blocks, for the preparation of protein-recognizing MIPs only one to three different functional monomers are typically used [12]. This is a limitation as compared with protein-based receptors. Furthermore, the limited conformational stability of proteins restricts the MIP-synthesis to aqueous conditions where hydrogen bonds are almost inefficient and hydrophobic interactions are likely to be more significant. Uncharged functional monomers have been most widely used for protein MIPs, because electrostatic binding may cause non-specific interactions with charged constituents of the sample [14].

### 2.2. Preparation of MIPs for Proteins

#### 2.2.1. Bulk Imprinting of Proteins

The bulk synthesis method, which is well established in synthesis of MIPs for recognition of low-molecular-weight compounds, is hardly applicable to macromolecules due to their hindered mobility in the highly reticulated polymeric networks. In the worst scenario the macromolecules become entrapped in the polymeric material with both their removal and rebinding prohibited. Nevertheless, “bulk imprinted” hydrogels based on acrylamide or agarose with low density of cross-linking and large pores have been developed as the precursors of MIPs for the recognition of proteins [44,45].

#### 2.2.2. Surface Imprinting

The essential prerequisite of macromolecular imprinting to create accessible binding sites amenable for free target-exchange between the MIP and the sample phase has been fulfilled by controlling the binding site generation solely at the surface of the polymer, i.e., surface imprinting. This is generally accomplished by creating the binding sites within highly cross-linked polymer films (a characteristic of the classical bulk imprinting, which ensures stability of the binding sites). However, the thickness of this MIP layer is extremely thin, i.e., comparable with the hydrodynamic radius of the protein so that only partial embedding of the template in the polymer occurs and complete entrapment is avoided. Such nanofilms will allow then the effective removal and rebinding of the target analyte. Certainly, there are many ways of realizations of such surface imprinted polymer films largely determined by the solid support used, orientation of the target, type of polymerization and functional monomers as well as by geometrical constrictions.

It is important to note that surface imprints are obviously also formed during bulk polymerization, but generally at low surface densities and large heterogeneities. Therefore, the distinctive feature of surface imprinting is the utmost control of the imprinting process, in terms of localization, and reproducible fabrication of homogeneous binding sites most often with large surface densities. In fact, to maximize the binding capacity of surface imprinted polymers the surface/volume ratio needs to be increased and therefore the formation of surface imprinted nanoparticles and nanostructures comes as a natural necessity to fulfill these expectations.

**Figure 5:**
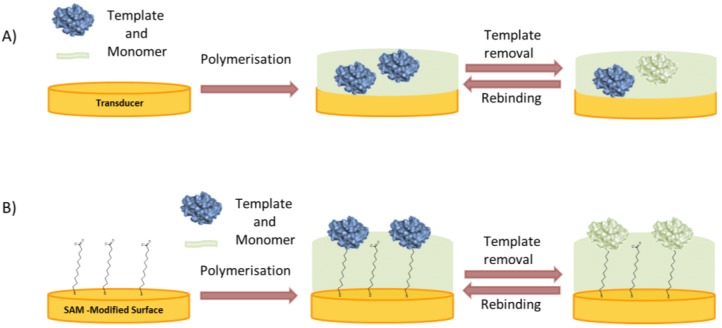
Surface imprinting approaches for the synthesis of MIP films for selective recognition of proteins: (**A**) Polymerization of a mixture of protein and monomer; (**B**) Binding of the protein to a self-assembled anchor layer for oriented immobilization of the protein prior to polymerization.

In the simplest approach an ultra-thin layer is formed on the surface of micro or nanoparticles or directly on the transducer by polymerizing a mixture of the template and monomer (Figure 5A) [46].

On the other hand, suspension, emulsion, or precipitation polymerization, which leads to the formation of micro- or nanobeads; MIP nanomaterials such as nanoparticles, nanospheres and MIP nanomaterial composites, have been applied [47,48,49]. These “Nano-MIPs” offer pronounced advantages in respect to increased binding capacity and also selective binding of biomacromolecules.

Furthermore, the protein was adsorbed on a planar surface, e.g., of mica or silicon, which is used to “stamp” a soft polymer layer for the production of protein-imprinted films (micro-contact imprinting) [50,51,52].

The homogeneity of the binding sites can be in principle increased if the template is immobilized in an oriented manner to the solid support prior to polymerization. Additionally, exploitation of a few but strong interactions at defined sites of the protein molecule with specifically designed monomers can enhance the imprinting effect. This approach was pioneered by the group of Mosbach. They used the complex formation between surface-exposed histidine residues of RNase A and a chelator at the surface of silica beads in the presence of Cu(II). However, the lack of three-dimensional cavities of a polymer will allow unhindered non-specific binding of other proteins with exposed histidine residues [10].

- In the “top-down” approach of surface imprinting the target protein is attached to a support or mold, which is removed after the formation of the polymer layer. Stereochemically complementary cavities are left behind on the inner surfaces [53,54,55].
- In the “bottom-up” concept the protein is immobilized directly on the substrate where the polymer is to be deposited (Figure 5B). Various interactions can be exploited to anchor the protein to the surface in an oriented manner, thereby ensuring the formation of uniform binding pockets. Glycoproteins can be immobilized by boronic acids [56,57] enzymes with their inhibitors or substrates [58,59] and, in general, any reversible receptor can be used as anchor, e.g., aptamers [60].
- The preparation of MIPs for the recognition of proteins does not necessarily need the use of the whole macromolecule as template, but a representative fragment may suffice (epitope approach). Consequently, imprinting a peptide sequence, which represents a small and exposed part of the whole protein, was successfully applied for MIPs which recognize the parent protein [61,62,63,64,65]. This approach has several advantages, e.g., the polymerization can be performed in organic solvents, the cost of the peptide is lower than for the protein, it can be synthesized in a very pure form and also a well selected peptide sequence may increase selectivity. On the other hand, the epitopes are typically built up from amino acids located in different sections of the peptide chain held in spatial proximity by the protein’s tertiary structure. A linear epitope, suitable for imprinting, can only be found at the exposed termini of the protein. Therefore, larger fragments of the protein have been also used as the template during polymerization, e.g., the Fab-fragment for a MIP towards IgG [66].

### 2.3. Binding Performance of MIPs

The most important parameters characterizing the performance of MIPs are the affinity and selectivity of target rebinding which determine the dynamic concentration range including the limit of detection and the cross reactivity. Binding of low molecular weight targets to the MIP in non-aqueous solvents is dominated by hydrogen bonds [12,15]. Furthermore, the target molecule is usually completely embedded in the MIP (“bulk imprinting”) resulting in strong interaction with the surrounding polymer. On the other hand, the imprinting of proteins requires aqueous conditions where hydrogen bonds are almost inefficient and hydrophobic interactions are likely to be more significant. In addition, the film thickness of surface imprinted layers is smaller than the characteristic dimension of the target in order to ensure effective exchange with the sample solution. Thus, interaction with the polymer is restricted to a fraction of the macromolecular target and affinity constants for non-covalent MIPs could hardly reach the sub-nanomolar region [12].

Many attempts have been made to enhance the performance of protein-MIPs by using different nanostructures on the surface prior to polymer deposition. Among them, the application of graphene [67] and carbon nanotubes [68] dominate. In addition to their excellent mechanical properties and electrical conductivity, they provide a large surface for the creation of binding sites.

The imprinting factor (IF), which is defined as the ratio of the signals of the MIP and the non-imprinted polymer (NIP) after rebinding of the target, reflects the ratio of specific binding of the MIP to the nonspecific binding at the NIP surface. At saturation concentrations it represents the ratio of binding capacities to the imprinted and non-imprinted polymer. The imprinting factor is influenced by the method, which was used to measure the target rebinding to the MIP and NIP. For surface plasmon resonance (SPR) and quartz crystal microbalance (QCM), the signal can be influenced by structural changes of the polymer. The frequently used current measurement given by the permeability of the MIPs to a redox marker—that is inversely proportional with the amount of target bound—reflects, not only the occupancy of the cavities by the target, but also the formation of “nonspecific” pores, which are only permeable for the redox marker [69,70,71,72]. Therefore, the conditions of template removal are crucial. The evaluation of the enzymatic activity [59] or of direct electron transfer, applicable however only for particular targets, is considerably more specific and gives a “functional” imprinting factor.

## 3. Preparation and Performance of Aptamers for the Recognition of Proteins

### 3.1. Preparation of Aptamers

Aptamers are primarily generated using the SELEX procedure, an iterative in vitro selection, in which aptamers are isolated from a random library of at least 10^14^ different nucleic acids using a targeted in vitro evolution [73]. In the classical aptamer selection techniques [74,75,76], the target is immobilized to a solid surface, for instance by Ni-NTA or streptavidin, for an optimal separation of non-binding and target binding molecules. It is important to emphasize that for selection of highly selective aptamers the protein targets need to be very pure and that may require expression of properly tagged proteins (e.g., His tag) to enable both their purification and controlled immobilization [77,78]. The magnetic particle-based SELEX is one of the core methods, owing in particular to the additional possibility of automation, which allows the reproducible and parallelized aptamer selection, even for multiple targets reducing the selection time. Several automated SELEX procedures have been developed [79,80,81,82,83,84]. The classical SELEX methodologies were further complemented with different techniques, such as capillary electrophoresis-based SELEX [85] and whole cell-SELEX [86,87], as well as their automation [88].

### 3.2. Binding Performance of Aptamers

Aptamers have various advantages compared to antibodies, which result in a broad field of applications [89,90,91]. It is important to emphasize that the aim of the in vitro selection process is solely to determine the sequence of the aptamers and not their production. Once their sequence established, aptamers can be inexpensively synthesized and reproduced. This is a major advantage with respect to MIPs where the imprinting process is directly used for production, i.e., the template is required all along for the MIP synthesis. Furthermore, the custom synthesis of nucleic acid aptamers enables their chemical modification for finely tuned physical chemical properties, stabilization and increased functionality. In particular, the in vivo stability and the half-life of aptamers can be enhanced by the chemical modification of nucleotides [92] or conjugation of molecules like polyethylene glycol (PEG) [93]. The integration of reporter groups like fluorescent dyes or biotin at well-defined sites of the aptamer makes them useful in various sensing applications. Furthermore, the smaller size of aptamers (<30 kDa) compared to antibodies enables a higher packing density on sensor surfaces and their in vivo delivery by tissue penetration. The long-term stability of aptamers allows easy storage and delivery and aptamers can recover their active conformation after denaturation. Their variety of structures results in often-higher specificity to a target compared with antibodies. Additionally, the generation of aptamers can be realized against toxic and non-immunogenic targets because of the pure in vitro process without the need of animals or in vivo conditions.

Due to these advantages, aptamers are used particularly in the expanding field of aptamer-based biosensors [94,95,96], termed aptasensors [97], or in other analytical tools like lateral flow devices for diagnostic [98], food [75] and environmental [90,99] analysis. Additionally, the therapeutic field offers further applications of aptamers [100,101,102,103]. Aptamers are utilized as imaging [104,105] and therapeutic agents, in target validation [106] and in regenerative medicine [107]. Furthermore, two special aspects should be mentioned. The activity of aptamer drugs can be controlled by complementary sequences (antidotes) because of their ability to interrupt the aptamer-target binding complex [108,109]. Furthermore, the artificial L-ribonucleic acid aptamers, so-called Spiegelmers [110,111], are highly resistant to degradation by nucleases and have several drug candidates in advanced phases of clinical research along with emerging diagnostic applications [92]. Despite all their advantages, the general perception of the aptamers’ performance as compared with that of antibodies is more critical. While the success of the aptamer selection is by definition probabilistic, the lack of tuning the selection process by considering the analytical application may also be detrimental for the performance of the selected aptamers. Ideally, the selection should be performed at the same ionic strength and pH as of the sample and very importantly counterselections steps should be included during selection to remove aptamers from the library that bind other constituents of the sample matrix. Aptamer generation was dramatically increased by the recent development of new SELEX techniques using alternative nucleic acid libraries of chemically modified nucleobases directly in the in vitro selection process. Even the main deficiency of not featuring hydrophobic moieties has been addressed by the implementation of SOMAmers, a new generation of high affinity aptamers for diagnostic and biomarker discovery [112,113,114]. Additionally, nucleotides modified by click reaction enable successful in vitro selection against certain targets, which could not be used successfully in former SELEX processes [115,116].

## 4. Catalytically Active MIPs and Aptamers

### 4.1. Catalytically Active MIPs

Molecularly imprinted polymers have not only been designed for binding of the analyte but also as enzyme mimics. In analogy with catalytically active antibodies (abzymes), stable analogues of the postulated transition state (TSA) of the catalyzed reaction are used as the template to mimic the active center of the enzyme [4,117]. This concept is appropriate for the preparation of hydrolase-like MIPs; however, the specific activity is several orders of magnitude lower as compared with that of esterase enzymes [118]. The first example of a MIP catalyst which showed a higher catalytic activity than the respective catalytic antibodies was realized using phosphate or phosphonate as TSA in the presence of an amidinium containing monomer and Zn^2+^ for the catalysis of carbonate hydrolysis [117,119].

In order to mimic redox enzymes, metal ions or metal complexes have been integrated into the polymer matrix of MIPs. Copper-containing MIPs could mimic the active site of tyrosinase by oxidizing catechol in the presence of atmospheric oxygen [120] and of nitroreductase in the electrocatalytic reduction of metronidazole [121]. A mimic of the seleno-enzyme glutathione peroxidase is based on 3-hydroxypropyl telluride [122] as the catalytic center, which is combined with the catalytic triad of the enzyme. In addition, redox-active groups of oxidoreductases (e.g., heme [123] or flavine [124] analogues) have been used in MIPs for the oxidation of 2,4-dichlorophenol [125], 5-hydroxyindole-3-acetamide [126] and homovalinic acid as the catalytic center.

### 4.2. Catalytically Active Aptamers: Aptazymes and Riboswitches

Aptamers have occasionally versatile catalytic effects in specific arrangements. Particularly, aptamers, used as therapeutic agents, or intramers, intracellularly expressed aptamers, have an effect on different biological pathways by binding to their targets [127].

Furthermore, riboswitches are short regulatory segments of a messenger RNA (mRNA), which regulate gene expression by binding of metabolites (small molecules) [128,129]. The best known riboswitches are found in bacteria [130], and also in plant and fungi [131]. The existence of natural regulatory aptamers (riboswitches) was discovered by the observations of homologies between RNA sequences in the 5′-untranslated region of transcripts and in vitro selected RNA aptamers [132]. The binding of the metabolite by an aptamer sequence induces a modulation of the ribosomal binding site (RBS) by a structural change of the proximal expression platform, which regulates the specific gene expression. Nowadays, many types of riboswitches are known, such as Lysine, Glycine, Purine, S-adenosylhomocysteine (SAH), S-adenosyl methionine (SAM), thiamin pyrophosphate (TPP) riboswitches, etc. [133].

Additionally, the combination of aptamers and ribozymes (ribonucleic acid enzymes) results in chimeric molecules, so-called aptazymes, consisting of an aptamer domain and a distant ribozyme module. These oligonucleotide-regulated ribozymes work like allosteric enzymes in which the catalytic activity is regulated by the binding of the aptamer target like a small molecule, a protein or another oligonucleotide [127,134,135]. Many aptazyme-based sample applications have developed in the field of biotechnology, diagnostics and therapeutics in the recent years. The elucidation of structures of aptamer-target complexes and ribozymes by NMR [136,137] and crystal [138,139,140] structure analysis enables the design of various aptazymes in which the binding of a target by an aptamer is normally connected with helix stabilization or refolding of the aptamers. Firstly, Breaker and co-workers developed an aptazyme [141,142] based on the adenosine triphosphate binding (ATP-binding) aptamer and the hammerhead (HH) ribozyme [143]. In the presence of ATP or adenosine, the cleavage rate of the ribozyme was decreased 180-fold compared to control reactions with other nucleoside triphosphates (NTPs) or deoxyadenosine triphosphate (dATP). The flavin mononucleotide (FMN) RNA aptamer was connected with stem II of HH ribozyme over a short randomized stem. The following in vitro selections have delivered variants with the potential of activation or inhibition of the HH cleavage activity in the presence of FMN [144,145]. Furthermore, aptazymes can be used as reporter ribozymes for screening processes in biosensors. For example, an adenosine diphosphate (ADP) aptamer based allosteric ribozyme was generated to monitor the pERK2 protein kinase activity in the presence of different drug-like compounds [146], whereas a protein-dependent ribozyme was utilized for affinity screening of the HIV-1 reverse transcriptase (RT) to a large library of 2500 compounds [147]. In addition, further aptazymes are based on a wide range of ribozymes like the hairpin ribozyme [148,149], the ligase ribozyme [150,151] or the Diels-Alder ribozyme [152,153].

## 5. Chemical Sensing Schemes

After their sequence established nucleic acid aptamers are produced by routine chemical synthesis, which offers utmost versatility in tuning their structure for chemical sensing applications. Thus the recognition sequence can be complemented with various functionalities, such as to enable straightforward immobilization to sensor transducers or nanoparticles, as well as with probes/reporters for signal generation, e.g., fluorescent and redox probes [154]. Aptamer-based protein sensing benefits additionally also from the broad methodological basis of nucleic acid assays, with many of the methods being inherent to nucleic acids, e.g., molecular beacons, polymerase chain reactions, etc. Thus, a large number of aptamer-based fluorescent detection methods were designed involving folding-refolding of aptamer strands [155], strand assembly-disassembly [156,157], as well as a variety of displacement assays. On the analogy of the fluorescent molecular beacons electrochemical detection of proteins can be enabled by using redox reporters incorporated in aptamer strands [158]. In this case, the aptamers are immobilized on an electrode surface and the electron transfer between the electrode and the redox active probe is modulated by the protein binding, i.e., the distance over which the charge transfer occurs is changed by the binding. Without entering deeper in the plethora of aptamer-based protein sensing schemes [159] the use of nucleic acid amplification schemes, which are again distinct properties of nucleic acid aptamers, is definitely worth mentioning. These schemes take advantage of the detection of the specifically bound nucleic acid aptamer strand or its designed elongations to implement it directly or indirectly as a template for polymerase chain reaction [160,161], rolling circle amplification [162], proximity ligation assay [163], etc. Thus in terms of the resourcefulness of sensing schemes aptamers have a clear advantage over MIPs, but this does not mean that MIPs lack “unique” transduction schemes. MIPs are materials with adjustable dimensions and properties that were exploited in sensing, e.g., growing photonic protein imprinted polymers by colloidal templating allows label-free quantitation of target proteins via recording the shift in their stop band upon protein biding [164]. Similarly, surface imprinted polymer nanofilms enable the label-free electrochemical sensing of target binding through the modulating effect on the permeability of these films [165].

In addition to the particular sensing schemes the general approaches that are implemented in other affinity assays, e.g., in immunosensors and DNA chips, were seamlessly overtaken for these biomimetic receptors. However, their surface confinement, interfacing with transducers and mechanism of action show many interesting features and challenges.

At the beginning of the analytical applications of MIPs and aptamers, both analytical reactors in flow systems [166,167] and lateral flow devices in the test strip format [168,169] have been described. For the realization of a biomimetic sensor the analyte recognition by the MIP or aptamer should be in close proximity to the surface of the signal generating transducer. Therefore, the aptamer has to be immobilized directly at the surface. MIP sensors mostly apply “surface imprinted” layers directly on the electrode, QCM-crystal or SPR-chip. Most often, the following schemes are applied:

- Label-free evaluation of changes of the recognition layer upon binding of the target protein by QCM and SPR

Both methods are especially appropriate for the detection of macromolecules whilst the measurement of low-molecular weight substances has low sensitivity. For SPR [46,58] and QCM [53,54,58,170] the signal reflects overall changes of refractive index or mass of the recognition layer, which can be induced not only by the target but also by nonspecific adsorption especially by electrostatic interactions with the negatively charged aptamer skeleton. For MIPs changes of the polymer layer swelling or shrinking can mask the binding event.

- Direct indication of target binding

Direct detection of the template protein’s characteristic absorption bands in Raman [171] and FTIR spectroscopy [172] offers the indication of the protein binding to the biomimetic recognition element. For electroactive or intrinsically fluorescent proteins and certain enzymes, the direct electron transfer [173] or fluorescence [174] and the assessment of enzymatic activity [59], respectively, offer a selective means for the detection of the protein binding to the MIP. If the target protein does not possess such properties, the rebinding can be selectively measured by applying a fluorescent or enzyme label on the target [54,63]. Binding of the analyte to labeled aptamers—called molecular beacons—can induce drastic conformational changes, which result in an enormous electrochemical signal or of fluorescence [175].

- Fluorescence quenching of the MIP layer upon protein binding

Fluorescent particles can be incorporated in the MIP [176] or fluorophores can be coated onto it [177] to make the polymer fluorescent. This fluorescence is then gradually quenched by binding of the target protein in increasing concentrations. Similarly to the above electrochemical methods, low protein concentrations are difficult to measure; therefore the limit of detection is generally in the low micromolar range.

- Changes in the MIP film permeability for an electro-active electrochemical probe

A frequently applied indirect method for the characterization of analyte binding to aptamers or MIPs is based on measuring cyclic voltammetry (CV) or electrochemical impedance (EI) spectra. It reflects the permeability of the recognition layer for a redox marker, e.g., ferrocyanide or ferrocene carboxylic acid [69,70,71,72]. According to the simplified mechanism for MIPs in the absence of the target protein, the empty binding sites permit the access of the redox marker to the underlying electrode surface while the protein binding will decrease the permeability in a concentration dependent manner. Problems may arise from the spontaneous adsorption of surface-active constituents of real samples at the electrode surface, which will overlay the effect of the target binding to the aptamer or the MIP cavities. A disadvantage of the method is that at low target concentrations minute decreases in the current are to be detected in a large base current. Nevertheless, several papers describe MIP and aptamer sensors for both low and high molecular weight targets with measuring ranges over several orders of magnitude and with sub-nanomolar lower limit of detection [69,71].

## 6. Conclusions and Outlook

In spite of the shorter history of aptamers the spectrum of analytes and the number of publications are comparable with that for MIPs. The highly reproducible production of “monoclonal” aptamers by chemical synthesis is a clear advantage whilst the preparation of MIPs typically leads to polymers with a distribution of different binding sites and differences between the batches of preparations. Progress is expected from an enhanced functional monomer library combined either with a rational design or with an empirical approach associated with high-throughput synthesis and detection platforms. As compared with antibodies and aptamers, MIPs have been developed only for a restricted spectrum of proteins and almost half of the published papers still use hemoglobin, serum albumins and avidin as model templates. Detection of marker proteins for cardiovascular disease [67,71], cancer [46,178], Alzheimer’s disease (AChE) [59] or virus infections are the prospective aims in the generation of MIPs. In spite of several reports claiming applicability [71], MIPs need still substantial improvement to reach the required sensitivity and to overcome disturbances caused by constituents of real samples.

As compared with antibodies or MIPs, the exchange rate of the aptamer-target complex is considerably higher. Furthermore, the direct readout of the bound analyte is easier since the integration of signal generating labels is well established. Both features make aptamers to potent candidates for in vivo sensing.

Because their large-scale production is claimed to be cheaper than the preparation of antibodies, MIPs are expected to be used as recognition elements in the decentralized medical diagnostics. For both aptamers and MIPs, the measurement of groups of chemically similar substances (group effects) or alternatively the indication of different analytes by only one recognition species would give additional advantages in comparison with the biological pendants.

## References

[1] Polyakov M.V. (1931). Adsorption properties and structure of silica gel. Zhurnal Fizieskoj Khimii/Akad. SSSR.

[2] Polyakov M.V., Kuleshina L., Neimark I. (1937). On the dependence of silica gel adsorption properties on the character of its porosity. Zhurnal Fizieskoj Khimii/Akad. SSSR.

[3] Dickey F.H. (1949). The preparation of specific adsorbents. Proc. Natl. Acad. Sci. USA.

[4] Wulff G., Sarhan A. (1972). Use of polymers with enzyme-analogous structures for resolution of racemates. Angew. Chem. Int. Ed..

[5] Wulff G., Vesper W., Grobe-Einsler R., Sarhan A. (1977). Enzyme-analogue built polymers, 4. On the synthesis of polymers containing chiral cavities and their use for the resolution of racemates. Makromol. Chem. Macromol. Chem. Phys..

[6] Arshady R., Mosbach K. (1981). Synthesis of substrate-selective polymers by host-guest polymerization. Macromol. Chem. Phys. Makromol. Chem..

[7] Glad M., Norrlow O., Sellergren B., Siegbahn N., Mosbach K. (1985). Use of silane monomers for molecular imprinting and enzyme entrapment in polysiloxane-coated porous silica. J. Chromatogr..

[8] Norrlow O., Glad M., Mosbach K. (1984). Acrylic polymer preparations containing recognition sites obtained by imprinting with substrates. J. Chromatogr..

[9] Uzun L., Turner A.P.F. (2016). Molecularly-imprinted polymer sensors: Realising their potential. Biosens. Bioelectron..

[10] Kempe M., Glad M., Mosbach K. (1995). An approach towards surface imprinting using the enzyme ribonuclease A. J. Mol. Recognit..

[11] Takeuchi T., Hishiya T. (2008). Molecular imprinting of proteins emerging as a tool for protein recognition. Org. Biomol. Chem..

[12] Whitcombe M.J., Chianella I., Larcombe L., Piletsky S.A., Noble J., Porter R., Horgan A. (2011). The rational development of molecularly imprinted polymer-based sensors for protein detection. Chem. Soc. Rev..

[13] Yang K., Zhang L., Liang Z., Zhang Y. (2012). Protein-imprinted materials: Rational design, application and challenges. Anal. Bioanal. Chem..

[14] Li S.J., Cao S.S., Whitcombe M.J., Piletsky S.A. (2014). Size matters: Challenges in imprinting macromolecules. Prog. Polym. Sci..

[15] Erdössy J., Horváth V., Yarman A., Scheller F.W., Gyurcsányi R.E. (2016). Electrosynthesized molecularly imprinted polymers for protein recognition. Trends Anal. Chem..

[16] Mipdatabase. http://www.mipdatabase.com.

[17] Ellington A.D., Szostak J.W. (1990). In vitro selection of RNA molecules that bind specific ligands. Nature.

[18] Robertson D.L., Joyce G.F. (1990). Selection in vitro of an RNA enzyme that specifically cleaves single-stranded DNA. Nature.

[19] Tuerk C., Gold L. (1990). Systematic evolution of ligands by exponential enrichment: RNA ligands to bacteriophage T4 DNA polymerase. Science.

[20] Bock L.C., Griffin L.C., Latham J.A., Vermaas E.H., Toole J.J. (1992). Selection of single-stranded DNA molecules that bind and inhibit human thrombin. Nature.

[21] Cohen B.A., Colas P., Brent R. (1998). An artificial cell-cycle inhibitor isolated from a combinatorial library. Proc. Natl. Acad. Sci. USA.

[22] Crawford M., Woodman R., Ko Ferrigno P. (2003). Peptide aptamers: Tools for biology and drug discovery. Brief. Funct. Genom. Proteom..

[23] Hoppe-Seyler F., Crnkovic-Mertens I., Tomai E., Butz K. (2004). Peptide aptamers: Specific inhibitors of protein function. Curr. Mol. Med..

[24] Reverdatto S., Burz D.S., Shekhtman A. (2015). Peptide aptamers: Development and applications. Curr. Top. Med. Chem..

[25] Breaker R.R. (2004). Natural and engineered nucleic acids as tools to explore biology. Nature.

[26] Feigon J., Dieckmann T., Smith F.W. (1996). Aptamer structures from a to zeta. Chem. Biol..

[27] Klussmann S. (2006). The Aptamer Handbook: Functional Oligonucleotides and Their Applications.

[28] Ellington A.D., Szostak J.W. (1992). Selection in vitro of single-stranded DNA molecules that fold into specific ligand-binding structures. Nature.

[29] Boiziau C., Toulme J.J. (2001). A method to select chemically modified aptamers directly. Antisense Nucleic Acid Drug Dev..

[30] Connell G.J., Illangesekare M., Yarus M. (1993). Three small ribooligonucleotides with specific arginine sites. Biochemistry.

[31] Famulok M. (1994). Molecular recognition of amino-acids by RNA-aptamers—An L-citrulline binding rna motif and its evolution into an L-arginine binder. J. Am. Chem. Soc..

[32] Wallis M.G., von Ahsen U., Schroeder R., Famulok M. (1995). A novel rna motif for neomycin recognition. Chem. Biol..

[33] Wochner A., Menger M., Orgel D., Cech B., Rimmele M., Erdmann V.A., Glokler J. (2008). A DNA aptamer with high affinity and specificity for therapeutic anthracyclines. Anal. Biochem..

[34] Mendonsa S.D., Bowser M.T. (2005). In vitro selection of aptamers with affinity for neuropeptide y using capillary electrophoresis. J. Am. Chem. Soc..

[35] Green L.S., Jellinek D., Jenison R., Ostman A., Heldin C.H., Janjic N. (1996). Inhibitory DNA ligands to platelet-derived growth factor b-chain. Biochemistry.

[36] Chen F., Zhou J., Luo F., Mohammed A.B., Zhang X.L. (2007). Aptamer from whole-bacterium selex as new therapeutic reagent against virulent mycobacterium tuberculosis. Biochem. Biophys. Res. Commun..

[37] Hamula C.L., Zhang H., Guan L.L., Li X.F., Le X.C. (2008). Selection of aptamers against live bacterial cells. Anal. Chem..

[38] Wang J., Jiang H., Liu F. (2000). In vitro selection of novel rna ligands that bind human cytomegalovirus and block viral infection. RNA.

[39] Shangguan D., Li Y., Tang Z.W., Cao Z.H.C., Chen H.W., Mallikaratchy P., Sefah K., Yang C.Y.J., Tan W.H. (2006). Aptamers evolved from live cells as effective molecular probes for cancer study. Proc. Natl. Acad. Sci. USA.

[40] Tang Z.W., Parekh P., Turner P., Moyer R.W., Tan W.H. (2009). Generating aptamers for recognition of virus-infected cells. Clin. Chem..

[41] Nimjee S.M., Rusconi C.P., Sullenger B.A. (2005). Aptamers: An emerging class of therapeutics. Annu. Rev. Med..

[42] Saffhill R., Schneider-Bernloehr H., Orgel L.E., Spiegelman S. (1970). In vitro selection of bacteriophage q-beta ribonucleic acid variants resistant to ethidium bromide. J. Mol. Biol..

[43] Scopus. https://www.scopus.com.

[44] Hjerten S., Liao J.L., Nakazato K., Wang Y., Zamaratskaia G., Zhang H.X. (1997). Gels mimicking antibodies in their selective recognition of proteins. Chromatographia.

[45] Tong D., Hetenyi C., Bikadi Z., Gao J.P., Hjerten S. (2001). Some studies of the chromatographic properties of gels (‘artificial antibodies/receptors’) for selective adsorption of proteins. Chromatographia.

[46] Bosserdt M., Erdőssy J., Lautner G., Witt J., Kohler K., Gajovic-Eichelmann N., Yarman A., Wittstock G., Scheller F.W., Gyurcsányi R.E. (2015). Microelectrospotting as a new method for electrosynthesis of surface-imprinted polymer microarrays for protein recognition. Biosens. Bioelectron..

[47] Ambrosini S., Beyazit S., Haupt K., Bui B.T.S. (2013). Solid-phase synthesis of molecularly imprinted nanoparticles for protein recognition. Chem. Commun..

[48] Pluhar B., Mizaikoff B. (2015). Advanced evaluation strategies for protein-imprinted polymer nanobeads. Macromol. Biosci..

[49] Zhou T.C., Zhang K., Kamra T., Bulow L., Ye L. (2015). Preparation of protein imprinted polymer beads by pickering emulsion polymerization. J. Mater. Chem. B.

[50] Hayden O., Lieberzeit P.A., Blaas D., Dickert F.L. (2006). Artificial antibodies for bioanalyte detection-sensing viruses and proteins. Adv. Funct. Mater..

[51] Lin H.Y., Hsu C.Y., Thomas J.L., Wang S.E., Chen H.C., Chou T.C. (2006). The microcontact imprinting of proteins: The effect of cross-linking monomers for lysozyme, ribonuclease a and myoglobin. Biosens. Bioelectron..

[52] Sener G., Ozgur E., Rad A.Y., Uzun L., Say R., Denizli A. (2013). Rapid real-time detection of procalcitonin using a microcontact imprinted surface plasmon resonance biosensor. Analyst.

[53] Bognar J., Szucs J., Dorko Z., Horvath V., Gyurcsanyi R.E. (2013). Nanosphere lithography as a versatile method to generate surface-imprinted polymer films for selective protein recognition. Adv. Funct. Mater..

[54] Menaker A., Syritski V., Reut J., Opik A., Horvath V., Gyurcsanyi R.E. (2009). Electrosynthesized surface-imprinted conducting polymer microrods for selective protein recognition. Adv. Mater..

[55] Lautner G., Kaev J., Reut J., Opik A., Rappich J., Syritski V., Gyurcsányi R.E. (2011). Selective artificial receptors based on micropatterned surface-imprinted polymers for label-free detection of proteins by spr imaging. Adv. Funct. Mater..

[56] Liu S.Q., Bakovic L., Chen A.C. (2006). Specific binding of glycoproteins with poly(aniline boronic acid) thin film. J. Electroanal. Chem..

[57] Wang S.S., Ye J., Bie Z.J., Liu Z. (2014). Affinity-tunable specific recognition of glycoproteins via boronate affinity-based controllable oriented surface imprinting. Chem. Sci..

[58] Dechtrirat D., Gajovic-Eichelmann N., Bier F.F., Scheller F.W. (2014). Hybrid material for protein sensing based on electrosynthesized mip on a mannose terminated selfassembled monolayer. Adv. Funct. Mater..

[59] Jetzschmann K.J., Jágerszki G., Dechtrirat D., Yarman A., Gajovic-Eichelmann N., Gilsing H.D., Schulz B., Gyurcsányi R.E., Scheller F.W. (2015). Vectorially imprinted hybrid nanofilm for acetylcholinesterase recognition. Adv. Funct. Mater..

[60] Jolly P., Tamboli V., Harniman R.L., Estrela P., Allender C.J., Bowen J.L. (2016). Aptamer-mip hybrid receptor for highly sensitive electrochemical detection of prostate specific antigen. Biosens. Bioelectron..

[61] Bossi A.M., Sharma P.S., Montana L., Zoccatelli G., Laub O., Levi R. (2012). Fingerprint-imprinted polymer: Rational selection of peptide epitope templates for the determination of proteins by molecularly imprinted polymers. Anal. Chem..

[62] Cenci L., Anesi A., Busato M., Guella G., Bossi A.M. (2016). Molecularly imprinted polymers coupled to matrix assisted laser desorption ionization mass spectrometry for femtomoles detection of cardiac troponin I peptides. J. Mol. Recognit..

[63] Dechtrirat D., Jetzschmann K.J., Stocklein W.F.M., Scheller F.W., Gajovic-Eichelmann N. (2012). Protein rebinding to a surface-confined imprint. Adv. Funct. Mater..

[64] Nishino H., Huang C.S., Shea K.J. (2006). Selective protein capture by epitope imprinting. Angew. Chem. Int. Ed..

[65] Rachkov A., Minoura N. (2000). Recognition of oxytocin and oxytocin-related peptides in aqueous media using a molecularly imprinted polymer synthesized by the epitope approach. J. Chromatogr. A.

[66] Erturk G., Uzun L., Tumer M.A., Say R., Denizli A. (2011). Fab fragments imprinted spr biosensor for real-time human immunoglobulin g detection. Biosens. Bioelectron..

[67] Wang X.D., Dong J., Ming H.M., Ai S.Y. (2013). Sensing of glycoprotein via a biomimetic sensor based on molecularly imprinted polymers and graphene-au nanoparticles. Analyst.

[68] Chen H.J., Zhang Z.H., Xie D., Cai R., Chen X., Liu Y.N., Yao S.Z. (2012). Surface-imprinting sensor based on carbon nanotubes/graphene composite for determination of bovine serum albumin. Electroanalysis.

[69] Cai D., Ren L., Zhao H., Xu C., Zhang L., Yu Y., Wang H., Lan Y., Roberts M.F., Chuang J.H. (2010). A molecular-imprint nanosensor for ultrasensitive detection of proteins. Nat. Nanotechnol..

[70] Cieplak M., Szwabinska K., Sosnowska M., Bikram K.C.C., Borowicz P., Noworyta K., D’Souza F., Kutner W. (2015). Selective electrochemical sensing of human serum albumin by semi-covalent molecular imprinting. Biosens. Bioelectron..

[71] Karimian N., Turner A.P.F., Tiwari A. (2014). Electrochemical evaluation of troponin T imprinted polymer receptor. Biosens. Bioelectron..

[72] Silva B.V.M., Rodriguez B.A.G., Sales G.F., Sotomayor M.D.T., Dutra R.F. (2016). An ultrasensitive human cardiac troponin t graphene screen-printed electrode based on electropolymerized-molecularly imprinted conducting polymer. Biosens. Bioelectron..

[73] Klug S.J., Famulok M. (1994). All you wanted to know about selex. Mol. Biol. Rep..

[74] Szeitner Z., András J., Gyurcsányi R.E., Mészáros T. (2014). Is less more? Lessons from aptamer selection strategies. J. Pharm. Biomed. Anal..

[75] Wu J., Zhu Y., Xue F., Mei Z., Yao L., Wang X., Zheng L., Liu J., Liu G., Peng C. (2014). Recent trends in selex technique and its application to food safety monitoring. Mikrochim. Acta.

[76] Yuce M., Ullah N., Budak H. (2015). Trends in aptamer selection methods and applications. Analyst.

[77] Balogh Z., Lautner G., Bardoczy V., Komorowska B., Gyurcsányi R.E., Mészáros T. (2010). Selection and versatile application of virus-specific aptamers. FASEB J..

[78] Lautner G., Balogh Z., Bardoczy V., Meszáros T., Gyurcsányi R.E. (2010). Aptamer-based biochips for label-free detection of plant virus coat proteins by spr imaging. Analyst.

[79] Drolet D.W., Jenison R.D., Smith D.E., Pratt D., Hicke B.J. (1999). A high throughput platform for systematic evolution of ligands by exponential enrichment (selex). Comb. Chem. High Throughput Screen..

[80] Cox J.C., Ellington A.D. (2001). Automated selection of anti-protein aptamers. Bioorg. Med. Chem..

[81] Eulberg D., Buchner K., Maasch C., Klussmann S. (2005). Development of an automated in vitro selection protocol to obtain rna-based aptamers: Identification of a biostable substance p antagonist. Nucleic Acids Res..

[82] Wochner A., Cech B., Menger M., Erdmann V.A., Glokler J. (2007). Semi-automated selection of DNA aptamers using magnetic particle handling. Biotechniques.

[83] Glokler J., Schutze T., Konthur Z. (2010). Automation in the high-throughput selection of random combinatorial libraries—Different approaches for select applications. Molecules.

[84] Hunniger T., Wessels H., Fischer C., Paschke-Kratzin A., Fischer M. (2014). Just in time-selection: A rapid semiautomated selex of DNA aptamers using magnetic separation and beaming. Anal. Chem..

[85] Hamedani N.S., Muller J. (2016). Capillary electrophoresis for the selection of DNA aptamers recognizing activated protein c. Methods Mol. Biol..

[86] Daniels D.A., Chen H., Hicke B.J., Swiderek K.M., Gold L. (2003). A tenascin-c aptamer identified by tumor cell selex: Systematic evolution of ligands by exponential enrichment. Proc. Natl. Acad. Sci. USA.

[87] Ohuchi S. (2012). Cell-selex technology. BioRes. Open Access.

[88] Hung L.Y., Wang C.H., Hsu K.F., Chou C.Y., Lee G.B. (2014). An on-chip cell-selex process for automatic selection of high-affinity aptamers specific to different histologically classified ovarian cancer cells. Lab Chip.

[89] Song K.M., Lee S., Ban C. (2012). Aptamers and their biological applications. Sensors (Basel).

[90] Tombelli S., Minunni M., Mascini M. (2007). Aptamers-based assays for diagnostics, environmental and food analysis. Biomol. Eng..

[91] Tsae P.K., DeRosa M.C. (2015). Outlook for aptamers after twenty five years. Curr. Top. Med. Chem..

[92] Szeitner Z., Lautner G., Nagy S.K., Gyurcsányi R.E., Mészáros T. (2014). A rational approach for generating cardiac troponin I selective spiegelmers. Chem. Commun..

[93] Pendergrast P.S., Marsh H.N., Grate D., Healy J.M., Stanton M. (2005). Nucleic acid aptamers for target validation and therapeutic applications. J. Biomol. Tech..

[94] Kim Y.S., Gu M.B. (2014). Advances in aptamer screening and small molecule aptasensors. Adv. Biochem. Eng. Biotechnol..

[95] Kim Y.S., Raston N.H., Gu M.B. (2016). Aptamer-based nanobiosensors. Biosens. Bioelectron..

[96] MacKay S., Wishart D., Xing J.Z., Chen J. (2014). Developing trends in aptamer-based biosensor devices and their applications. IEEE Trans. Biomed. Circuits Syst..

[97] Van den Kieboom C.H., van der Beek S.L., Mészáros T., Gyurcsányi R.E., Ferwerda G., de Jonge M.I. (2015). Aptasensors for viral diagnostics. TrAC Trends Anal. Chem..

[98] Ozalp V.C., Kavruk M., Dilek O., Bayrac A.T. (2015). Aptamers: Molecular tools for medical diagnosis. Curr. Top. Med. Chem..

[99] Strehlitz B., Reinemann C., Linkorn S., Stoltenburg R. (2012). Aptamers for pharmaceuticals and their application in environmental analytics. Bioanal. Rev..

[100] Ray P., Viles K.D., Soule E.E., Woodruff R.S. (2013). Application of aptamers for targeted therapeutics. Arch. Immunol. Ther. Exp. (Warsz.).

[101] Wochner A., Menger M., Rimmele M. (2007). Characterisation of aptamers for therapeutic studies. Exp. Opin. Drug Discov..

[102] Zhou J., Rossi J.J. (2009). The therapeutic potential of cell-internalizing aptamers. Curr. Top. Med. Chem..

[103] Zhu G., Ye M., Donovan M.J., Song E., Zhao Z., Tan W. (2012). Nucleic acid aptamers: An emerging frontier in cancer therapy. Chem. Commun. (Camb.).

[104] Gijs M., Aerts A., Impens N., Baatout S., Luxen A. (2016). Aptamers as radiopharmaceuticals for nuclear imaging and therapy. Nucl. Med. Biol..

[105] Jin C., Zheng J., Li C., Qiu L., Zhang X., Tan W. (2015). Aptamers selected by cell-selex for molecular imaging. J. Mol. Evol..

[106] Blank M., Blind M. (2005). Aptamers as tools for target validation. Curr. Opin. Chem. Biol..

[107] Guo K.T., SchAfer R., Paul A., Gerber A., Ziemer G., Wendel H.P. (2006). A new technique for the isolation and surface immobilization of mesenchymal stem cells from whole bone marrow using high-specific DNA aptamers. Stem Cells.

[108] Liu X., Cao G., Ding H., Zhang D., Yang G., Liu N., Fan M., Shen B., Shao N. (2004). Screening of functional antidotes of rna aptamers against bovine thrombin. FEBS Lett..

[109] Rusconi C.P., Roberts J.D., Pitoc G.A., Nimjee S.M., White R.R., Quick G., Scardino E., Fay W.P., Sullenger B.A. (2004). Antidote-mediated control of an anticoagulant aptamer in vivo. Nat. Biotechnol..

[110] Vater A., Klussmann S. (2015). Turning mirror-image oligonucleotides into drugs: The evolution of spiegelmer(®) therapeutics. Drug Discov. Today.

[111] Vater A., Klussmann S. (2003). Toward third-generation aptamers: Spiegelmers and their therapeutic prospects. Curr. Opin. Drug Discov. Dev..

[112] Brody E.N., Gold L., Lawn R.M., Walker J.J., Zichi D. (2010). High-content affinity-based proteomics: Unlocking protein biomarker discovery. Exp. Rev. Mol. Diagn..

[113] Ochsner U.A., Green L.S., Gold L., Janjic N. (2014). Systematic selection of modified aptamer pairs for diagnostic sandwich assays. Biotechniques.

[114] Webber J., Stone T.C., Katilius E., Smith B.C., Gordon B., Mason M.D., Tabi Z., Brewis I.A., Clayton A. (2014). Proteomics analysis of cancer exosomes using a novel modified aptamer-based array (somascan) platform. Mol. Cell. Proteom..

[115] Tolle F., Brandle G.M., Matzner D., Mayer G. (2015). A versatile approach towards nucleobase-modified aptamers. Angew. Chem. Int. Ed. Engl..

[116] Tolle F., Rosenthal M., Pfeiffer F., Mayer G. (2016). Click reaction on solid phase enables high fidelity synthesis of nucleobase-modified DNA. Bioconjug. Chem..

[117] Wulff G., Liu J.Q. (2012). Design of biomimetic catalysts by molecular imprinting in synthetic polymers: The role of transition state stabilization. Acc. Chem. Res..

[118] Robinson D.K., Mosbach K. (1989). Molecular imprinting of a transition-state analog leads to a polymer exhibiting esterolytic activity. J. Chem. Soc. Chem. Commun..

[119] Liu J.Q., Wulff G. (2004). Molecularly imprinted polymers with strong carboxypeptidase a-like activity: Combination of an amidinium function with a zinc-ion binding site in transition-state imprinted cavities. Angew. Chem. Int. Ed..

[120] Lakshmi D., Bossi A., Whitcombe M.J., Chianella I., Fowler S.A., Subrahmanyam S., Piletska E.V., Piletsky S.A. (2009). Electrochemical sensor for catechol and dopamine based on a catalytic molecularly imprinted polymer-conducting polymer hybrid recognition element. Anal. Chem..

[121] Gu Y., Yan X.Y., Li C., Zheng B., Li Y.R., Liu W.L., Zhang Z.Q., Yang M. (2016). Biomimetic sensor based on molecularly imprinted polymer with nitroreductase-like activity for metronidazole detection. Biosens. Bioelectron..

[122] Huang X., Yin Y.Z., Liu Y., Bai X.L., Zhang Z.M., Xu J.Y., Shen J.C., Liu J.Q. (2009). Incorporation of glutathione peroxidase active site into polymer based on imprinting strategy. Biosens. Bioelectron..

[123] Diaz-Diaz G., Dineiro Y., Menendez M.I., Blanco-Lopez M.C., Lobo-Castanon M.J., Miranda-Ordieres A.J., Tunon-Blanco P. (2011). Molecularly imprinted catalytic polymers with biomimetic chloroperoxidase activity. Polymer.

[124] Sode K., Ohta S., Yanai Y., Yamazaki T. (2003). Construction of a molecular imprinting catalyst using target analogue template and its application for an amperometric fructosylamine sensor. Biosens. Bioelectron..

[125] Zhang J., Lei J.P., Ju H.X., Wang C.Y. (2013). Electrochemical sensor based on chlorohemin modified molecularly imprinted microgel for determination of 2,4-dichlorophenol. Anal. Chim. Acta.

[126] Antuna-Jimenez D., Blanco-Lopez M.C., Miranda-Ordieres A.J., Lobo-Castanon M.J. (2014). Artificial enzyme with magnetic properties and peroxidase activity on indoleamine metabolite tumor marker. Polymer.

[127] Famulok M., Hartig J.S., Mayer G. (2007). Functional aptamers and aptazymes in biotechnology, diagnostics, and therapy. Chem. Rev..

[128] Tucker B.J., Breaker R.R. (2005). Riboswitches as versatile gene control elements. Curr. Opin. Struct. Biol..

[129] Vitreschak A.G., Rodionov D.A., Mironov A.A., Gelfand M.S. (2004). Riboswitches: The oldest mechanism for the regulation of gene expression?. Trends Genet..

[130] Nudler E., Mironov A.S. (2004). The riboswitch control of bacterial metabolism. Trends Biochem. Sci..

[131] Bocobza S.E., Aharoni A. (2008). Switching the light on plant riboswitches. Trends Plant Sci..

[132] Borsuk P., Dzikowska A., Empel J., Grzelak A., Grzeskowiak R., Weglenski P. (1999). Structure of the arginase coding gene and its transcript in aspergillus nidulans. Acta Biochim. Pol..

[133] Edelmann C.M. (1989). Clinical quiz. Renal tubular acidosis (rta). Pediatr. Nephrol..

[134] Burgstaller P., Jenne A., Blind M. (2002). Aptamers and aptazymes: Accelerating small molecule drug discovery. Curr. Opin. Drug Discov. Dev..

[135] Famulok M. (2005). Allosteric aptamers and aptazymes as probes for screening approaches. Curr. Opin. Mol. Ther..

[136] Fan P., Suri A.K., Fiala R., Live D., Patel D.J. (1996). Molecular recognition in the fmn-rna aptamer complex. J. Mol. Biol..

[137] Patel D.J. (1997). Structural analysis of nucleic acid aptamers. Curr. Opin. Chem. Biol..

[138] Mir A., Golden B.L. (2016). Two active site divalent ions in the crystal structure of the hammerhead ribozyme bound to a transition state analogue. Biochemistry.

[139] Oberthur D., Achenbach J., Gabdulkhakov A., Buchner K., Maasch C., Falke S., Rehders D., Klussmann S., Betzel C. (2015). Crystal structure of a mirror-image l-rna aptamer (spiegelmer) in complex with the natural l-protein target ccl2. Nat. Commun..

[140] Russo Krauss I., Merlino A., Giancola C., Randazzo A., Mazzarella L., Sica F. (2011). Thrombin-aptamer recognition: A revealed ambiguity. Nucleic Acids Res..

[141] Tang J., Breaker R.R. (1997). Rational design of allosteric ribozymes. Chem. Biol..

[142] Tang J., Breaker R.R. (1998). Mechanism for allosteric inhibition of an atp-sensitive ribozyme. Nucleic Acids Res..

[143] Scott W.G., Horan L.H., Martick M. (2013). The hammerhead ribozyme: Structure, catalysis, and gene regulation. Prog. Mol. Biol. Transl. Sci..

[144] Araki M., Okuno Y., Hara Y., Sugiura Y. (1998). Allosteric regulation of a ribozyme activity through ligand-induced conformational change. Nucleic Acids Res..

[145] Soukup G.A., Breaker R.R. (1999). Design of allosteric hammerhead ribozymes activated by ligand-induced structure stabilization. Structure.

[146] Srinivasan J., Cload S.T., Hamaguchi N., Kurz J., Keene S., Kurz M., Boomer R.M., Blanchard J., Epstein D., Wilson C. (2004). Adp-specific sensors enable universal assay of protein kinase activity. Chem. Biol..

[147] Hartig J.S., Famulok M. (2002). Reporter ribozymes for real-time analysis of domain-specific interactions in biomolecules: Hiv-1 reverse transcriptase and the primer-template complex. Angew. Chem. Int. Ed. Engl..

[148] Appel B., Marschall T., Strahl A., Muller S. (2012). Kinetic characterization of hairpin ribozyme variants. Methods Mol. Biol..

[149] Najafi-Shoushtari S.H., Mayer G., Famulok M. (2004). Sensing complex regulatory networks by conformationally controlled hairpin ribozymes. Nucleic Acids Res..

[150] Kossen K., Vaish N.K., Jadhav V.R., Pasko C., Wang H., Jenison R., McSwiggen J.A., Polisky B., Seiwert S.D. (2004). High-throughput ribozyme-based assays for detection of viral nucleic acids. Chem. Biol..

[151] Robertson M.P., Ellington A.D. (2000). Design and optimization of effector-activated ribozyme ligases. Nucleic Acids Res..

[152] Helm M., Petermeier M., Ge B., Fiammengo R., Jaschke A. (2005). Allosterically activated diels-alder catalysis by a ribozyme. J. Am. Chem. Soc..

[153] Serganov A., Keiper S., Malinina L., Tereshko V., Skripkin E., Hobartner C., Polonskaia A., Phan A.T., Wombacher R., Micura R. (2005). Structural basis for diels-alder ribozyme-catalyzed carbon-carbon bond formation. Nat. Struct. Mol. Biol..

[154] Radi A.-E., Acero Sánchez J.L., Baldrich E., O’Sullivan C.K. (2006). Reagentless, reusable, ultrasensitive electrochemical molecular beacon aptasensor. J. Am. Chem. Soc..

[155] Cao Z., Tan W. (2005). Molecular aptamers for real-time protein-protein interaction study. Chem.—Eur. J..

[156] Yamamoto R., Kumar P.K.R. (2000). Molecular beacon aptamer fluoresces in the presence of tat protein of HIV-1. Genes Cells.

[157] Nutiu R., Li Y. (2003). Structure-switching signaling aptamers. J. Am. Chem. Soc..

[158] Xiao Y., Lubin A.A., Heeger A.J., Plaxco K.W. (2005). Label-free electronic detection of thrombin in blood serum by using an aptamer-based sensor. Angew. Chem. Int. Ed..

[159] Cho E.J., Lee J.-W., Ellington A.D. (2009). Applications of aptamers as sensors. Annu. Rev. Anal. Chem..

[160] Zhang H., Wang Z., Li X.-F., Le X.C. (2006). Ultrasensitive detection of proteins by amplification of affinity aptamers. Angew. Chem. Int. Ed..

[161] Fischer N.O., Tarasow T.M., Tok J.B.H. (2008). Protein detection via direct enzymatic amplification of short DNA aptamers. Anal. Biochem..

[162] Yang L., Fung C.W., Cho E.J., Ellington A.D. (2007). Real-time rolling circle amplification for protein detection. Anal. Chem..

[163] Fredriksson S., Gullberg M., Jarvius J., Olsson C., Pietras K., Gustafsdottir S.M., Ostman A., Landegren U. (2002). Protein detection using proximity-dependent DNA ligation assays. Nat. Biotech..

[164] Hu X., Li G., Huang J., Zhang D., Qiu Y. (2007). Construction of self-reporting specific chemical sensors with high sensitivity. Adv. Mater..

[165] Yoshimi Y., Ohdaira R., Iiyama C., Sakai K. (2001). “gate effect” of thin layer of molecularly-imprinted poly(methacrylic acid-co-ethyleneglycol dimethacrylate). Sens. Actuators B Chem..

[166] Sartori L.R., Santos W.D.R., Kubota L.T., Segatelli M.G., Tarley C.R.T. (2011). Flow-based method for epinephrine determination using a solid reactor based on molecularly imprinted poly(fepp-maa-egdma). Mater. Sci. Eng. C-Mater. Biol. Appl..

[167] Reddy S.M., Sette G., Phan Q. (2011). Electrochemical probing of selective haemoglobin binding in hydrogel-based molecularly imprinted polymers. Electrochim. Acta.

[168] Chen A., Yang S. (2015). Replacing antibodies with aptamers in lateral flow immunoassay. Biosens. Bioelectron..

[169] Zhou W., Huang P.J., Ding J., Liu J. (2014). Aptamer-based biosensors for biomedical diagnostics. Analyst.

[170] Tretjakov A., Syritski V., Reut J., Boroznjak R., Opik A. (2016). Molecularly imprinted polymer film interfaced with surface acoustic wave technology as a sensing platform for label-free protein detection. Anal. Chim. Acta.

[171] Lv Y., Qin Y., Svec F., Tan T. (2016). Molecularly imprinted plasmonic nanosensor for selective sers detection of protein biomarkers. Biosens. Bioelectron..

[172] Moreira F.T.C., Sharma S., Dutra R.A.F., Noronha J.P.C., Cass A.E.G., Sales M.G.F. (2014). Protein-responsive polymers for point-of-care detection of cardiac biomarker. Sens. Actuators B-Chem..

[173] Bosserdt M., Gajovic-Eichelman N., Scheller F.W. (2013). Modulation of direct electron transfer of cytochrome c by use of a molecularly imprinted thin film. Anal. Bioanal. Chem..

[174] Zhao W.T., Chen Z.H., Xue B., Sun L.Q., Luo A.Q. (2011). A biomimetic sensor for fast lysozyme detection. Adv. Mater. Res..

[175] Sefah K., Phillips J.A., Xiong X., Meng L., Van Simaeys D., Chen H., Martin J., Tan W. (2009). Nucleic acid aptamers for biosensors and bio-analytical applications. Analyst.

[176] Yang Y.Q., He X.W., Wang Y.Z., Li W.Y., Zhang Y.K. (2014). Epitope imprinted polymer coating cdte quantum dots for specific recognition and direct fluorescent quantification of the target protein bovine serum albumin. Biosens. Bioelectron..

[177] Deng Q., Wu J., Zhai X., Fang G., Wang S. (2013). Highly selective fluorescent sensing of proteins based on a fluorescent molecularly imprinted nanosensor. Sensors.

[178] Feng X.B., Gan N., Zhou J., Li T.H., Cao Y.T., Hu F.T., Yu H.W., Jiang Q.L. (2014). A novel dual-template molecularly imprinted electrochemiluminescence immunosensor array using ru(bpy)(3)(2+)-silica@poly-l-lysine-au composite nanoparticles as labels for near-simultaneous detection of tumor markers. Electrochim. Acta.

